# Artificial intelligence revolutionizing anesthesia management: advances and prospects in intelligent anesthesia technology

**DOI:** 10.3389/fmed.2025.1571725

**Published:** 2025-08-06

**Authors:** Yannan Cao, Yixin Wang, Hang Liu, Lei Wu

**Affiliations:** ^1^School of Medicine, The Second Affiliated Hospital, Zhejiang University, Hangzhou, China; ^2^The Faculty of Medicine, Dalian University of Technology, Dalian, China

**Keywords:** anesthesia, artificial intelligence, perioperative anesthesia management, machine learning, neural networks

## Abstract

With the development of artificial intelligence (AI), AI-related technologies are being applied in many fields of medicine. Anesthesia is now widely used in surgery, emergency resuscitation, pain treatment and other fields. However, different from some other common biomedical signals, such as the electrocardiogram (ECG), electroencephalogram (EEG), and some other medical imaging or biomarkers could be easily processed and analyzed by AI-related models, how to collect the relevant data in the anesthesia process is still a challenge, that has led to little current work on combining AI and anesthesia. However, it can be foreseen that the combination of AI and anesthesia will become increasingly important. This paper presents a comprehensive review of anesthesia with AI based methods which have been now used in the preoperative phase, intraoperative phase, and postoperative phase. We first overview some crucial concepts of artificial intelligence, then discuss the related applications of artificial intelligence used in different phases of the anesthesia period, finally, we look forward to the future development of intelligent anesthesia. We hope through this review, we can provide comprehensive and objective guidance in AI-related anesthesia process to help anesthesiologists use more advanced AI techniques to diagnose and treat patients during the anesthesia period.

## 1 Introduction

Artificial Intelligence (AI) is a broad field focused on developing systems capable of performing tasks that typically require human intelligence. With advancements in hardware and algorithms, AI applications have expanded beyond research labs, impacting areas like speech recognition, image classification, and medical diagnostics (1). In the medical field, machine learning (ML) and deep learning (DL) are commonly used. Techniques like convolutional neural networks (CNNs) and support vector machines (SVMs) can automatically learn from data. For example, they can analyze MRI or CT images to help detect and classify diseases such as tumors or cardiovascular problems (2). These models help doctors by offering quick and data-based insights, reducing the time needed for diagnosis and improving accuracy. AI demonstrates significant value in healthcare, extending beyond diagnostic assistance to play a pivotal role in optimizing therapeutic processes. A prime example is in intensive care units (ICUs), where delirium is frequently associated with suboptimal sedation management, and conventional approaches often struggle to balance between oversedation and undersedation. In anesthesiology, AI technologies enable real-time analysis of patients' physiological parameters and medical histories, achieving not only precise anesthetic dosing and intelligent intraoperative monitoring but also predicting potential complications and optimizing personalized medication regimens, thereby significantly enhancing perioperative safety (3). With continuous technological advancements, AI is driving the field of anesthesiology toward greater precision and personalization.

**Figure 1:**
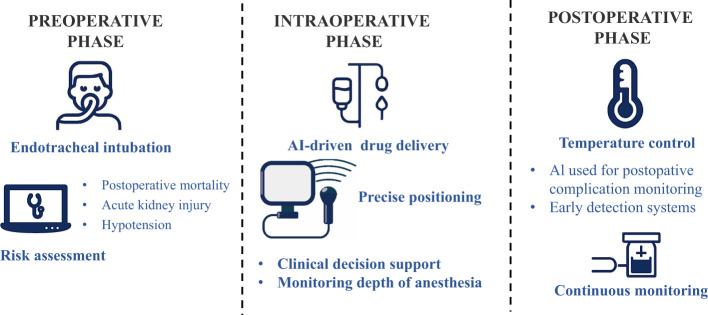
An overview of the use of AI in anesthesia.

The innovative application of AI in anesthesiology is systematically transforming perioperative management systems. By leveraging multimodal data fusion analysis, AI technologies have established an end-to-end solution spanning the pre-intra-postoperative continuum: (1) Preoperatively, machine learning algorithms enable accurate identification of high-risk patients and prediction of postoperative complications (4); (2) Intraoperatively, real-time physiological signal analysis facilitates dynamic early warning of adverse events like hypotension (5); and (3) Postoperatively, AI optimizes rehabilitation monitoring. Particularly noteworthy is the reinforcement learning model developed by Lee et al. (6), which overcomes limitations of conventional sedation management through closed-loop control of dexmedetomidine administration, establishing the first patient-specific adaptive sedation protocol. These technological advances not only validate the clinical value of AI in enhancing anesthesia safety and efficiency (4, 7), but also signify the transition of anesthesiology into a new era of data-driven precision medicine. Our review employs a narrative literature analysis methodology to investigate research advancements and clinical translation of AI technologies in anesthesiology. As shown in Figure 1, the research team has constructed a three-dimensional framework for intelligent perioperative anesthesia, encompassing temporal dimensions (preoperative risk assessment → intraoperative real-time regulation → postoperative outcome prediction), and application dimensions (personalized medication → complication prediction → resource optimization), providing a systematic analysis through these interconnected perspectives.

## 2 Method

This review explores how artificial intelligence and related technologies are used during different stages of surgery in clinical anesthesia. The focus lies in evaluating current research progress in this area. A literature search was performed using the PubMed database with combinations of keywords such as “artificial intelligence,” “machine learning,” “deep learning,” “anesthesia,” and “anesthesiology” (56). The inclusion criteria encompassed all peer-reviewed, English-language articles published between March 2021 and March 2025, including original research and narrative reviews. We excluded studies involving animals, editorials, letters to the editor, and abstracts. Additionally, we manually reviewed the reference lists of the included papers, incorporating any relevant studies that met the inclusion criteria Table 1.

**Table 1:** PubMed search strategy (2021–2025).

**Step 1-3: AI-related terms**
**Query #1:** “Artificial Intelligence”[All Fields] OR “AI”[All Fields]
**Query #2:** “Machine Learning”[All Fields]
**Query #3:** “Deep Learning”[All Fields]
**Step 4-5: Combine with Anesthesia terms**
**Query #4:** #1 OR #2 OR #3
**Query #5:** “Anesthesia”[All Fields] OR “Anesthesia Management”[All Fields] OR “Perioperative Anesthesia”[All Fields]
**Query #6:** #4 AND #5
**Step 6-8: Time filter and exclusions**
**Query #7:** (“2021”[Date - Publication] : “2025”[Date - Publication])
**Query #8:** “Case Report”[Publication Type] OR “Conference Abstract”[Publication Type]
**Step 9-10: Final filter**
**Query #9:** #6 AND #7
**Query #10:** #9 NOT #8

## 3 AI and preoperative phase

The preoperative phase is essential for minimizing anesthesia-related risks and improving patient outcomes. Accurate risk prediction enables clinicians to identify vulnerable patients and anticipate complications such as hypotension, acute kidney injury, or postoperative mortality. Meanwhile, effective airway assessment is critical for planning intubation and avoiding intraoperative emergencies. Artificial intelligence supports both tasks by analyzing complex clinical data, improving prediction accuracy, and enhancing the safety and precision of anesthesia planning.

### 3.1 Risk prediction

The use of artificial intelligence by analyzing vast amounts of medical data, such as patient records, laboratory test results, and imaging data, can detect potential risk factors and disease characteristics, thereby aiding doctors in early risk assessment. This integration of technology can provide a more comprehensive understanding of each patient's unique medical history, leading to improved patient outcomes and overall quality of care (8). Applying AI in the pre-anesthesia phase can significantly enhance the understanding of a patient's health status and surgical risks. Soong et al. (9) developed a machine learning-based risk stratification model to predict 90-day mortality in patients with hepatocellular carcinoma undergoing liver resection. Using the XGBoost algorithm and structured national-level clinical data, the model achieved strong discriminative performance (AUROC = 0.9376). The AUROC (Area Under the Receiver Operating Characteristic Curve) is a standard metric used to evaluate the discriminatory ability of a binary classification model. An AUROC of 0.5 indicates no better than random performance, while a value of 1.0 represents perfect classification. Early prediction of acute kidney injury (AKI) following adult cardiac surgery enables prompt identification and intervention, thereby facilitating timely clinical decision-making. Zhang et al. (10) developed a risk predictor for AKI after liver transplantation using supervised machine learning techniques and visualized the underlying mechanisms to aid in clinical decision-making. Li et al. (11) used multicenter data and MIMIC-IV to develop a model. It outperformed 12 algorithms, including KNeighborsClassifier, with an AUROC of 0.85. Least absolute shrinkage and selection operator (LASSO) regression selected key preoperative factors, like blood urea nitrogen and serum creatinine. The Shapley Additive Explanations (SHAP) analysis improved model transparency, clarifying predictive factors. These advances enhance risk stratification in anesthesiology.

In the preoperative assessment stage, artificial intelligence technology assists in risk prediction and medical history information extraction, improving the accuracy and efficiency of anesthesia management. Bishara et al. (12) developed the Opal platform, a clinical machine learning system built on the Anesthesia Information Management System (AIMS). The platform integrates electronic health record (EHR) data and supports model visualization, feature extraction, and prediction. It collected preoperative data from 29,004 surgical patients and extracted 155 variables to build a postoperative acute kidney injury (AKI) prediction model. A gradient boosting tree algorithm was used, achieving an AUC of 0.85 on the test set. The model also demonstrated high sensitivity (0.9) and specificity (0.8), indicating strong clinical utility. Similarly, accurate preoperative prediction of red blood cell transfusion is essential for optimizing blood resource allocation, reducing unnecessary type and screen tests, and enhancing patient safety. Zapf et al. (13) developed a machine learning model for this purpose using EHR data. To address data imbalance, the study employed Synthetic Minority Oversampling Technique (SMOTE) and weighted sampling techniques. These methods improved model sensitivity and ensured better performance in detecting transfusion needs.

### 3.2 Airway management

Endotracheal intubation plays a vital role in patients undergoing general anesthesia, intensive care, or emergency intervention, particularly in situations such as surgery, trauma, or respiratory failure, and is a critical step in ensuring oxygenation and ventilation. However, difficult airway management remains a challenge and can lead to serious complications, including hypoxia, aspiration, and even death if not recognized in a timely manner. In high-risk departments such as intensive care, anesthesia, and emergency care, good preparedness is not only prevention, but also central to patient survival. The introduction of emerging technologies–such as ultrasonography, nasal endoscopy, and AI-assisted imaging–has provided more objective tools for airway assessment. However, differences in predictive accuracy across methods still remain. For complex or high-risk patients, relying on a single approach is insufficient. The key lies in integrating multiple assessment tools, applying individualized clinical judgment, and maintaining adaptability in the face of anatomical uncertainty (14). A CNN-based model trained on specific medical facial landmarks demonstrated strong robustness, with test loss consistently remaining below 0.01, indicating stable predictive performance (15). Hayasaka et al. (16) proposed a facial image-based intubation difficulty classification method with a sensitivity of 81.8%, a specificity of 83.3%, and an AUC of 0.864, indicating its feasibility for clinical application. A model incorporating attention mechanisms to extract discriminative deep features from oral images achieved a classification accuracy of 97.5% under five-fold cross-validation, further supporting the utility of deep learning in airway image classification (17). Shim et al. (18) developed a machine learning model based on elastic net regression to predict endotracheal tube depth in pediatric patients under 7 years old. The model uses an elastic net strategy that combines L1 and L2 regularization to reduce overfitting and improve interpretability. It requires only four basic preoperative variables–age, gender, height, and weight–as input. The target label is the endotracheal tube depth measured on chest radiographs. This modeling approach is simple, stable, and easily deployable, aligning well with the anesthesia field's emphasis on lightweight, clinically practical models.

## 4 AI and intraoperative phase

**Figure 2:**
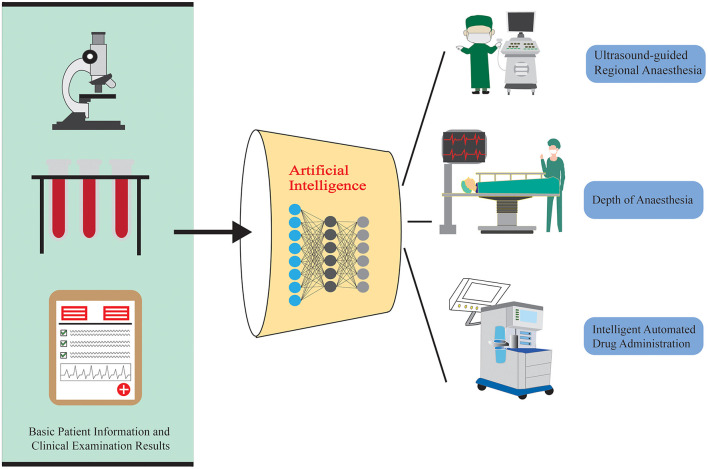
An application framework for the combination of intraoperative anesthesia AI.

When AI is combined with surgical anesthesia, it gives birth to a revolution in the medical field, pushing the surgical process into a new era of more precision and intelligence. Surgery, as a high-risk medical procedure, places exceptionally high demands on anesthesiologists. They must maintain absolute concentration and sensitivity during the operation to ensure the safety of the patient's life. AI brings new capabilities to this setting by enabling real-time monitoring, precision drug dosing, and enhanced imaging interpretation. As shown in Figure 2, the integration of anesthesia and artificial intelligence during surgery covers the following aspects.

### 4.1 Intelligent sedation and drug delivery

Intraoperative anesthesia management requires real-time adjustment of the patient's physiological parameters, such as anesthesia depth, blood pressure, heart rate, and respiratory function, to ensure surgical safety and stability. Accurate titration of anesthetic drugs is particularly important for maintaining optimal sedation and avoiding intraoperative awareness or hemodynamic instability. Artificial intelligence-based technologies, especially those using machine learning and reinforcement learning algorithms, are increasingly being used to automate and personalize sedation management. Reinforcement learning frameworks have also been enhanced by incorporating pharmacokinetic-pharmacodynamic (PK-PD) simulations, allowing models to retain accuracy even under complex conditions such as delays in drug concentration feedback and bispectral index (BIS) variability (19). Shenoy et al. (20) explored multiple regression-based machine learning algorithms–including neural networks, support vector machines, and Gaussian process regression–to predict remifentanil pharmacokinetics using patient-specific physiological parameters. Their optimized Gaussian process model achieved superior accuracy compared to conventional PK-PD models, demonstrating the feasibility of AI in real-time, individualized drug concentration forecasting. The results showed that Gaussian process regression (GPR) performed best among all models, with the lowest mean square error (RMSE = 5.40) and the highest coefficient of determination (R^2^ = 0.9616), and further improved the model performance through Bayesian optimization.

### 4.2 Monitoring depth of anesthesia and consciousness

During surgery, we often rely on multimodal monitoring tools, including electroencephalogram (EEG), electrocardiogram (ECG), blood pressure, heart rate, and end-tidal gas concentrations, to accurately assess the depth of anesthesia and ensure patient safety. To quantify both arousal and awareness dimensions of consciousness, Afshar et al. (21) employed a combinatorial deep learning model incorporating bidirectional long short-term memory (BiLSTM) and attention mechanisms, achieving 88.7% accuracy in real-time depth-of-anesthesia (DoA) classification despite imbalanced BIS value distributions. Building on this, Lee et al. (22) proposed the Explainable Consciousness Indicator (ECI), which applies convolutional neural networks (CNN) to time-series EEG data. In the other approach, Schmierer et al. (23) applied empirical wavelet transform (EWT) to EEG signals for DoA prediction. EEG provides direct insight into brain states, while other physiological signals provide complementary information. Yeh et al. (24) developed a portable ECG-monitoring prototype using Arduino and Raspberry Pi hardware, integrating a ResNet-based classifier for real-time waveform recognition. The system allowed remote access to ECG data, offering the potential for mobile cardiovascular risk monitoring before and during anesthesia. The SQI-DOANet model designed by Yu et al. (25) combined EEG signal quality evaluation and deep attention mechanism to achieve a predictive correlation of up to 0.88 on the VitalDB dataset, showing the practical application potential of AI models in complex intraoperative environments. Dussan et al. (26) and others built an anesthesia depth classification system based on multimodal signals from EEG, ECG, and NIBP, integrating central and autonomic nervous features. The neural network achieved the best classification performance when jointly using the complexity index of brain waves (CBI) and the heart rate variability index (CVI).

### 4.3 Ultrasound-guided regional anesthesia

Regional anesthesia relies on ultrasound guidance technology to achieve precise positioning to ensure that anesthetic drugs are accurately injected into specific nerves or tissue areas to achieve the desired anesthesia effect. Bowness et al. (27) analyzed the results of 40 ultrasound scans of seven different body areas by three regional anesthesia experts. They evaluated conventional ultrasound videos and videos enhanced with AI highlighting and found from the scoring results that the AI technology performed well in accurately identifying specific anatomical structures, achieving an accuracy of 99.7%. Subsequently, the research team conducted a multicenter external validation of the AI-assisted platform based on the ScanNav system, focusing on assessing its clinical applicability in ultrasound-guided regional nerve blocks. ScanNav is an AI image enhancement device designed to highlight anatomical structures in real-time B-mode ultrasound images. The system employs a U-Net based deep learning model that automatically identifies and labels key anatomical areas. In 720 clinical videos covering 9 block regions, the AI system achieved an anatomical recognition accuracy of 93.5%. Most clinical experts believe that the system's image enhancement feature helps reduce the risk of nerve damage and block failure. The study integrated dimensions such as image segmentation performance and expert evaluation, to reflect strong systematization and scalability (28, 29). Similarly, deep learning models have improved brachial plexus identification in ultrasound images, achieving a balance between segmentation accuracy and processing speed (30, 31). Integration of AI has also been proven to be valuable in challenging clinical scenarios. Compagnone et al. (32) evaluated a portable, handheld ultrasound device enhanced with artificial intelligence (Accuro^®^, Rivanna Medical, Charlottesville, VA, USA). The system integrated real-time ultrasound imaging with machine learning algorithms to automatically identify spinal anatomical landmarks. This AI-assisted device enabled clinicians to determine the optimal intervertebral insertion point and estimate the depth to the epidural space. The study focused on severely obese parturients (BMI = 64.5 kg/m^2^). Compared with traditional palpation and conventional ultrasound, the AI-enhanced approach significantly improved the first-attempt success rate of epidural catheter placement. In further research, Zhao et al. (33) constructed an artificial neural network (ANN) based on U-net, which achieved real-time automatic recognition of key anatomical structures in ultrasound images of thoracic paravertebral nerve block (TPVB).

### 4.4 Multiple monitoring and precise intervention

During gastrointestinal endoscopy, anesthesia is widely used because it can effectively relieve discomfort and ensure that the patient remains still, thus improving the success rate. ENDOANGEL is a computer-aided system that incorporates deep convolutional neural network technology and works in conjunction with traditional endoscopic equipment to provide real-time reminders for endoscopic surgeries. One of its main purposes is to assist the anesthesiologist in monitoring the patient's status more effectively. ENDOANGEL ensures a smooth examination process by sending reminders to the anesthesiologist about adding or stopping medications. The application of this technology helps improve the safety and efficiency of inspections (34). Xu et al. (35) building on previous work, evaluated the use of ENDOANGLE for anesthesia quality control in gastroenterology. An algorithm has been developed designed to continuously identify and analyze patients' vital signs from devices equipped with complex sensors to predict the occurrence of serious complications (36). Lofgren et al. (37) combined non-invasive urine oxygen monitoring with traditional preoperative indicators to develop a risk prediction model for intraoperative AKI. The study integrates real-time physiological signal collection, feature selection, and dynamic prediction. This aligns with current trends in anesthesia research, which emphasize both timeliness and system interoperability.

The integration of nociception monitors can be embedded within a closed-loop system to optimize the management of analgesic medications and identify patients more likely to experience severe pain postoperatively. This promises to promote more precise and personalized proactive pain intervention measures. An increasing number of monitors are being utilized to quantify patients' nociceptive responses during the anesthesia process, thereby providing a more accurate reflection of intraoperative stimulation. Recent studies have demonstrated the advantages of multimodal deep learning approaches in nociception monitoring. For instance, Abdel Deen et al. proposed a neural network architecture that combines electroencephalography (EEG), photoplethysmography (PPG), and electrocardiography (ECG) signals to predict nociceptive states using multilayer perceptrons (MLP) and long short-term memory (LSTM) models. Trained on expert-annotated data, their model achieved robust performance across key surgical events such as intubation, incision, and extubation, highlighting the value of integrating multiple physiological signals for enhanced intraoperative nociception assessment (38).

## 5 AI and postoperative phase

The postoperative period is a vulnerable period, especially for the elderly and high-risk patients, during which complications such as delirium, cardiac events, and deterioration of vital signs may occur. Early identification and intervention are key to improving recovery and outcomes. AI offers promising tools for predicting, detecting, and managing postoperative risks through continuous monitoring, data-driven risk scoring, and multimodal analysis.

### 5.1 Predicting postoperative delirium

Postoperative delirium, a transient mental state disturbance after surgery or medical treatment, is more common in elderly patients. It is generally considered to be of milder severity compared to other major postoperative complications (39, 40).

Zhao et al. (41) investigated postoperative delirium (POD) in elderly patients undergoing hip fracture surgery under spinal or general anesthesia. They analyzed perioperative features, including preoperative preparation time, frailty index, and intraoperative vasopressor use, achieving predictive accuracies of 83.67% to 87.75% using machine learning models trained on electronic anesthesia records. Röhr et al. (42) has successfully integrated electroencephalogram (EEG) data into a machine learning approach to provide a reliable assessment of POD risk. This multimodal approach substantially improved predictive accuracy (AUC increased from 0.75 to 0.80), underscoring the value of brain signal monitoring in real-time POD risk detection, particularly when stratified by anesthetic technique. Song et al. (43), on the other hand, emphasized model interpretability. By comparing six algorithms and applying SHAP analysis, the authors identified key biochemical markers such as Brain Natriuretic Peptide (BNP), C-reactive protein(CRP), and lactate dehydrogenase(LDH) as significant contributors to POD risk. This highlights the role of systemic inflammation and cardiac biomarkers in the pathogenesis of delirium, while also supporting clinical transparency in AI-driven prediction. Unlike prior models focusing primarily on clinical or physiological data, Wan et al. (44) incorporated blood-based biomarkers into POD prediction. Using lipid-related and inflammatory indicators–such as cholesterol, trimethylamine-N-oxide (TMAO), and IL-6–their model achieved comparable performance (AUC ~0.80) to physiology-driven models. The findings suggest that metabolic and immune pathways may contribute meaningfully to delirium risk and offer a novel direction for biomarker-informed prediction.

### 5.2 Vital sign monitoring

Postoperative deterioration often manifests subtly through changes in physiological signals before overt clinical symptoms arise. AI-enhanced monitoring systems, utilizing real-time data from wearable devices or bedside monitors, enable early detection of critical events such as hemodynamic instability or cardiac complications. A machine learning-driven screening tool known as the Cardiac Comorbidity Risk Score (CCoR) has been developed to identify patients at elevated risk for major adverse cardiac events (MACE) within four weeks of hip or knee arthroplasty (36). This screening tool represents a new advance in the field of artificial intelligence. Unlike traditional tools such as the Revised Cardiac Risk Index (RCRI), CCoR requires no additional testing and relies solely on existing diagnostic codes, achieving an area under the receiver operator characteristics curve (AUROC) of 80% in a large validation cohort (>445,000 patients), and demonstrating superior predictive power across sex, age, and comorbidity subgroups–even in patients without known RCRI conditions. In addition to static risk stratification, continuous vital sign monitoring offers dynamic insight into postoperative risk. Using wearable technology, Onishchenko et al. (45) collected real-time physiological data–including heart rate, respiratory rate, and blood pressure–to predict serious postoperative complications in high-risk patients. Among various machine learning models tested, random forest and boosted ensemble methods achieved the best balance between sensitivity and false positive rates. These findings underscore the potential of integrating continuous monitoring with AI algorithms to support timely clinical intervention and improve postoperative outcomes. Further expanding the scope of AI-based perioperative monitoring, Hoshijima et al. (46) applied gradient boosting models to predict postoperative nausea and vomiting (PONV) across 33,676 adult surgical cases. Intraoperative total blood loss emerged as the strongest risk factor, with additional contributors including female sex, limited fluid infusion, use of desflurane, and lateral positioning. Gradient boosting was chosen for its ability to model complex nonlinear relationships. It also handles heterogeneous perioperative data effectively and provides high predictive accuracy. The model achieved an AUC of 0.77, illustrating how machine learning can uncover complex intraoperative contributors to postoperative complications beyond hemodynamics alone. Body temperature is a key vital sign that reflects both physiological homeostasis and potential pathological changes. In the postoperative period, continuous temperature monitoring is particularly critical, as new-onset fever is often the earliest–and sometimes the only–clinical indicator of serious complications such as infections, thromboembolic events, or drug reactions (47). The patient's body temperature is one of the key vital signs and can accurately reflect the body's physiological status and metabolic activities. During the postoperative period, continuous monitoring of the patient's temperature is particularly important. After surgery, patients may be in a state of physical and psychological stress, which may lead to fluctuations in body temperature. Therefore, continuous body temperature monitoring helps medical staff detect and deal with potential complications promptly, ensuring a smooth patient recovery process (48).

## 6 Limitations and challenges

The development of artificial intelligence has attracted widespread attention and expectations. But sometimes, it also raises the myth that AI seems to be going to completely replace the role of the clinician. The concern that AI will replace clinicians is often overstated. While large models are increasingly implemented in hospitals, their primary function is to support clinicians in analysis and decision-making, not to substitute for clinical diagnosis (49). In current medical practice, AI applications are mainly concentrated in decision support and simulation. In anesthesiology, most professionals remain optimistic about AI-assisted tools and consider their predictive outputs to be a useful reference (50). In the field of medicine, especially in the critical field of anesthesia, innovation and technological advancements are crucial.

While advancing artificial intelligence technologies, researchers must proceed with caution. It is essential to continuously improve correction and fine-tuning mechanisms to ensure that task-specific models remain reliable and secure in broader clinical use (51). Although AI holds significant promise, its application in anesthesiology faces several key challenges. One major limitation is the narrow scope of available data. Many datasets are restricted to specific surgical procedures or patient subgroups, limiting the model's ability to generalize across diverse anesthesia scenarios. Although AI holds significant promise, its application in anesthesiology faces several key challenges. One major limitation is the narrow scope of available data. Many datasets are confined to specific surgical procedures or patient subgroups, which limits the model's ability to generalize across varied clinical scenarios. For instance, Hoshijima et al. (46) developed a machine learning model to predict postoperative nausea and vomiting (PONV) using data from over 33,000 adult patients at a single tertiary-care hospital. The dataset, although large in scale, was restricted to adult patients undergoing general anesthesia in a single academic center in Japan over a 10-year period. Pediatric patients, regional anesthesia cases, and outpatient procedures were excluded. This homogeneity in patient characteristics and surgical settings limits the external validity of the model, especially when applied to more diverse or high-risk populations. While the model demonstrated good predictive performance, its applicability to other anesthesia types, institutions, or more diverse populations remains uncertain. Since model performance depends heavily on both the volume and diversity of data, broader access to high-quality and representative datasets is essential to enhance the robustness and generalizability of AI applications in anesthesiology. Data heterogeneity is another concern. Patient information collected across different institutions often varies in format and quality, and may include considerable noise. When merging such datasets, data harmonization is necessary to maintain model accuracy (52).

In addition, anesthesia involves large volumes of sensitive patient data. Ensuring data security and patient privacy remains a major challenge (51). There is a need for compliant data management and transmission frameworks that can safeguard confidentiality in clinical AI applications. For a long time, artificial intelligence technology has been widely used in the healthcare sector. Part of the diagnostic evaluation is to extract patient clinical information, examination results, and other data from electronic health records (EHR), and perform model training and analysis. One of the challenges faced by artificial intelligence technology in data analysis is privacy leakage. In the healthcare system, EHR data is private. Even if metadata such as patient information is removed, more is needed to ensure the full protection of patient privacy. Especially in complex healthcare environments, public healthcare databases may be accessed by multiple parties, including hospitals, insurance companies (53).

## 7 Advantages and future development of combining artificial intelligence with anesthesia

With the continuous emergence of new technologies and methods, anesthesia is no longer isolated but closely connected with multiple fields to form a complex and diverse system. This intersection and integration bring unprecedented challenges to anesthesia, and also provide more opportunities for anesthesiologists and researchers to explore and practice. The research and application of AI in anesthesiology are becoming more and more extensive, and preliminary results have been achieved, which are mainly reflected in preoperative anesthesia management, intraoperative drug delivery, pain management, and postoperative complication prediction. AI can analyze large amounts of anesthesia records and clinical data through machine learning and deep learning algorithms to discover potential patterns and associations, and help anesthesiologists make more accurate diagnoses and predictions.

AI technology has significantly advanced the development of closed-loop anesthesia control systems, improving the stability and safety of the anesthesia process by continuously monitoring vital signs and automatically adjusting drug infusion parameters (54). Beyond operating rooms, office-based anesthesia (OBA) presents unique monitoring challenges due to lower-resource environments. Addressing this, Wang et al. (55) introduced Anes-Metanet, combining CNN and LSTM to extract power spectral density (PSD) features while modeling temporal dependencies. In a small validation cohort, the model achieved 81.8% accuracy in classifying three levels of consciousness: awake, semi-awake, and unconscious. In anesthesiology, from preoperative planning and preoperative assessment to intraoperative monitoring and postoperative management, artificial intelligence technology can profoundly impact all preoperative care phases, improving anesthesia outcomes, increasing surgical safety, and improving patient outcomes. The research and application of machine learning in the anesthesia discipline are becoming more and more extensive, and initial results have been achieved, mainly in preoperative anesthesia management, postoperative complication prediction, drug delivery, pain management, etc. Artificial intelligence can analyze large amounts of anesthesia records and clinical data through machine learning and deep learning algorithms, discover potential patterns and associations, and help anesthesiologists make more accurate diagnoses and predictions. We searched the literature at the intersection of artificial intelligence and anesthesia, intending to identify technologies in the field of artificial intelligence used in anesthesia research and their application in clinical practice in anesthesiology.

Artificial intelligence can analyze large amounts of anesthesia records and clinical data through machine learning and deep learning algorithms, discover potential patterns and associations, and help anesthesiologists make more accurate diagnoses and predictions. Artificial intelligence can control anesthesia equipment, adjust the anesthetic dose, and monitor the patient's indicators for improved accuracy and safety. Overall, the application of artificial intelligence in the field of anesthesia has broad prospects and is expected to bring a more efficient, safe, and personalized anesthesia experience to anesthesiologists and patients. However, with the development of artificial intelligence, a series of ethics, privacy, and security issues also need to be solved to ensure that the application of artificial intelligence can genuinely benefit human health.

## 8 Conclusion

With the vigorous development of artificial intelligence technologies such as deep learning and multi-modal analysis, the application of artificial intelligence technology in perioperative medicine has been continuously promoted. In the field of anesthesia, although the application of artificial intelligence is still in its infancy, a lot of progress has been made in recent years. Shortly, the combination of anesthesiology and artificial intelligence is bound to bring about major changes in perioperative medicine, and the work of anesthesiologists may change dramatically. More accurate, safer, and more effective anesthesia technology is worth looking forward to, bringing more efficient, comfortable, and safe medical effects and guarantees to patients.
